# Quality of life is associated with chronic inflammation in schizophrenia: a cross-sectional study

**DOI:** 10.1038/srep10793

**Published:** 2015-06-04

**Authors:** Faugere M., Micoulaud-Franchi J.A., Alessandrini M., Richieri R., Faget-Agius C., Auquier P., Lançon C., Boyer L.

**Affiliations:** 1 Department of Psychiatry, La Conception University Hospital, 13009 Marseille, France; 2 Aix-Marseille Univ., EA 3279 - Public Health, Chronic Diseases and Quality of Life - Research Unit, 13005 Marseille, France; 3 Department of Clinical Neurophysiology, Sleep Clinique, Pellegrin University Hospital, 33076 Bordeaux, France; 4 Bordeaux University, USR CNRS 3413 SANPSY, Research Unit, 33000 Bordeaux, France

## Abstract

Inflammation may play a crucial role in the pathogenesis of schizophrenia. However, the association between chronic inflammation and health outcomes in schizophrenia remains unclear, particularly for patient-reported outcomes. The aim of this study was to investigate the relationship between quality of life (QoL) and chronic inflammation assessed using C -Reactive Protein (CRP) in patients with schizophrenia. Two hundred and fifty six patients with schizophrenia were enrolled in this study. After adjusting for key socio-demographic and clinical confounding factors, patients with high levels of CRP (>3.0 mg/l) had a lower QoL than patients with normal CRP levels (OR = 0.97, 95% CI = 0.94–0.99). An investigation of the dimensions of QoL revealed that psychological well-being, physical well-being and sentimental life were the most salient features of QoL associated with CRP. Significant associations were found between lower educational level (OR = 4.15, 95% CI = 1.55–11.07), higher body mass index (OR = 1.16, 95% CI = 1.06–1.28), higher Fagerström score (OR = 1.22, 95% CI = 1.01–1.47) and high levels of CRP. After replications with longitudinal approaches, the association between QoL and chronic inflammation may offer interesting interventional prospects to act both on inflammation and QoL in patients with schizophrenia.

Schizophrenia occurs in approximately 1% of the population worldwide^1^. This disorder is a chronic disease characterised by psychotic symptoms^2^, cognitive impairment and functional decline^3^. All of these characteristics substantially impact the quality of life (QoL) of patients with schizophrenia^4^,^5^. Although there is strong evidence that multicomponent treatment involving medication, cognitive behavioural therapy, education, and exercise have beneficial effects on symptoms and functioning, most patients still have impaired QoL including affective, emotional, and social dimensions^6^,^7^. New approaches are therefore needed to act efficiently on QoL^8^.

Recent studies have explored questions about how non-biological factors, including affective, emotional, and social information, may be associated with chronic inflammation. In non-psychiatric populations, elevated C - Reactive Protein (CRP) has been reported to be associated with lower QoL level amongst the general population^9^, older individuals^10^ and in several chronic diseases (e.g., diabetes)^11^. The association between CRP and QoL may therefore offer interesting interventional prospects to act both on inflammation and QoL. These findings are of utmost importance in psychiatry, given that inflammatory responses and immune reactions may play a crucial role in the pathogenesis of schizophrenia^12^,^13^. Various studies have reported abnormalities of the immune reactions with the implication of blood lymphocyte abnormalities^12^, cytokine alterations^13^, oxidative stress anomalies^14^ and CRP elevations^15^ in schizophrenia. From a clinical perspective, elevated CRP levels are associated in schizophrenia with some pejorative psychiatric features^16^, high psychotic symptoms^17^,^18^ and high cognitive impairment^19^ . Moreover, elevated CRP levels are also associated with metabolic syndrome^20^ and high cardiovascular disease risk^21^. All of the clinical features that are associated with an elevated CRP level in schizophrenia are known to be associated with impaired QoL. Currently, however, no study has specifically investigated the relationship between impaired QoL and elevated CRP levels in patients with schizophrenia.

The aim of this study was to investigate the relationship between QoL and chronic inflammation assessed using CRP in patients with schizophrenia, in addition to considering key socio-demographic and clinical confounding factors.

## Methods

### Study participants

The study evaluated all prospective patients attending daytime hospital hours in our university and psychiatric hospital during a period of 4 years, from June 2010 to September 2014. The inclusion criteria were as follows: (1) age range 18–85 years old, (2) diagnosis of schizophrenia according to the Diagnostic and Statistical Manual of Mental Disorders, 4th edition (DSM-IV-TR) criteria^22^, (3) antipsychotic medication stable for a minimum of 3 months, and (4) French as a native language. The exclusion criteria were as follows: (1) diagnoses other than schizophrenia on Axis I of the DSM-IV-TR, except for nicotine dependence, (2) major non-psychiatric disease, (3) mental retardation and (4) any identifiable acute, intermittent or chronic infections or being on routine anti-inflammatory or immunosuppressive therapy: a clinical examination was performed, based on interrogatory about infectious signs and history, physical examination (including the following examination: temperature, weight, mouth, lymph nodes, abdomen, skin and cardiopulmonary auscultation) and serology (HIV, HBV and HCV). The data collection was approved by the Commission Nationale de l’Informatique et des Libertés (CNIL number 1223715). This study was constructed in accordance with the Declaration of Helsinki and French good clinical practices^23^. All of the patients were informed of the study and gave written, informed consent after a standardised and structured clinical interview.

### Data collection

The following data were collected:

1. Socio-demographic information: gender, age, educational level, professional activity.
2. Clinical characteristics: duration of disorder; body mass index (BMI); smoking status assessed by the Fagerström Test for Nicotine Dependence (FTND)^24^; alcohol consumption assessed by the Alcohol Use Disorders Identification Test (AUDIT)^25^; psychotic symptoms based on the Positive and Negative Syndrome Scale (PANSS), which consists of three subscales (positive, negative, and general psychopathology)^26^,^27^.
3. Drug information: antipsychotic medications (presence of atypical antipsychotic), presence of clozapine, presence of olanzapine, and chlorpromazine equivalent dose (in milligrams per day). Among atypical antipsychotics, clozapine and olanzapine are of specific interest for our study because of their potential link to metabolic syndrome and chronic inflammation^28^,^29^,^30^.
4. QoL measurement: QoL was assessed using the SQoL 18 questionnaire, which is a self-administered, multidimensional instrument developed and validated for the specific assessment of QoL in patients with schizophrenia^31^,^32^,^33^,^34^. The SQoL 18 consists of 18 items describing the following eight dimensions: Psychological Well-being (PsW), Self-Esteem (SE), Family Relationships (RFa), Relationships with Friends (RFr), Resilience (RE), Physical Well-being (PhW), Autonomy (AU), and Sentimental Life (SL) as well as a global score (the index)^33^. Dimensions and index scores range from 0, indicating the lowest QoL, to 100, the highest QoL.
5. Chronic inflammatory marker: serum levels of CRP were determined using sensitive regular immunoassays (ELISA). The results are expressed as milligram per litre. The detection limit was 0.08 μg/ml. Patients were classified into 2 groups: normal CRP level (≤3.0 mg/l) and High CRP (>3.0 mg/l)^21^,^35^,^36^.

### Statistical analysis

The characteristics of the entire group of patients with schizophrenia are expressed in proportions or as means and standard deviations. The socio-demographic and clinical characteristics, drug information, and QoL were compared between the two groups (normal CRP level vs. High CRP) using Student t test for continuous variables and Chi-square test for categorical variables.

A multivariate logistic regression was then performed to estimate the adjusted Odds Ratio (OR) and its corresponding 95% confidence interval (CI) for an association between the SQoL 18 index (independent variable) and CRP (dependent variable), with adjustment for confounding factors selected from the univariate based on a threshold p-value ≤0.20 (age, educational level, duration of disorder, BMI, Fagerström test and SQoL 18 index were selected). A set of additional variables was included in the models because of their clinical and socio-demographic interest (gender, presence of atypical antipsychotics, AUDIT, PANSS total score)^17^,^18^,^29^,^37^,^38^. This analysis was repeated replacing the SQoL 18 index with each dimension of the SQoL 18 to determine the most salient features of QoL associated with CRP.

All of the tests were two-sided. Statistical significance was defined as p < 0.05. Statistical analysis was performed using the SPSS version 18.0 software package (SPSS Inc., Chicago, IL, USA).

## Results

### Patient characteristics

Two hundred fifty six outpatients with schizophrenia participated in our study (Table 1). The mean age of the patients was 35.96 years (±12.01), and 71.9% of the patients were male. The patients showed moderate severity of symptoms, with a total PANSS score corresponding to 71.09 (±24.40) and sub-scores of 15.20 (±6.88), 20.00 (±7.95) and 35.89 (±12.09), respectively, for positive, negative, and general psychopathology factors. Of the total number of patients, the mean chlorpromazine equivalent dose was 856.69 (±791.44) milligrams per day; 88.3% were treated by atypical antipsychotics, 24.2% were treated by clozapine, and 15.7% were treated by olanzapine.

### Comparison of patients with normal CRP level and High CRP

Among the 256 patients with schizophrenia, 100 (39.06%) had a high CRP level. Compared to patients with normal CRP levels, those with high CRP levels were significantly older (p = 0.027), with a lower education level (p = 0.021), a longer duration of disorder (p = 0.018), a higher BMI (p = 0.000) and a higher Fagerström score (p = 0.041). Concerning QoL, patients with high CRP levels reported lower QoL scores for the SQoL 18 index, the PhW and the SL dimensions (p = 0.016, p = 0.005 and p = 0.003, respectively). A trend was observed for the SE, PsW and RE dimensions (p = 0.067, p = 0.076 and p = 0.073, respectively).

### Factors associated with High CRP

In the multivariate analyses reported in Tables 2 and 3, the relationship between the SQoL 18 index score and CRP level remained significant after adjusting for socio-demographic, clinical and drug characteristics. An educational level ≤12 years, a higher BMI and a higher Fagerström score remained significantly associated with high CRP level. An investigation of the various dimensions of the SQoL 18 revealed that PsW, PhW and SL were the most salient features of QoL associated with CRP. A trend was observed for the SE dimension.

## Discussion

This study investigated the relationship between QoL and chronic inflammation in patients with schizophrenia. Our findings provide evidence for a moderate association between high CRP levels and low QoL levels, including psychological well-being, physical well-being and sentimental life dimensions of QoL. Additionally, low educational level, high BMI and high Fagerström score also showed a significant relationship with high CRP level, whereas the severity of psychotic symptoms was not associated with CRP.

The main finding of our study is the existence of the relationship between QoL and chronic inflammation in patients with schizophrenia, after adjusting for key socio-demographic and clinical confounding factors. These findings support previous studies that reported the association between low QoL levels and high CRP levels amongst non-psychiatric populations^9^,^10^,^11^. However, the cross-sectional design of our study precludes any conclusions about the directionality of the association between QoL and inflammation in schizophrenia.

We hypothesise that QoL through its psychological, physical and social features predicts chronic inflammation in schizophrenia. Several studies reported that psychosocial factors, including poor well-being, were predictors of chronic inflammation and poor health^39^. The influence of psychological well-being on CRP may be supported by studies that reported an association between psychological stress and the dysregulation of the hypothalamic-pituitary-adrenal axis involved in inflammation^40^. Physical well-being may result from the physical fitness of individuals, which is also involved in inflammatory processes. Physically fit individuals demonstrate lower inflammatory responses to mental stress^41^. Moreover, regular exercise has been consistently associated with anti-inflammatory effects, whereas sedentary behaviour is now considered an important risk factor for inflammation and many health conditions independent of physical activity levels^42^. Concerning the relationship between sentimental life and CRP, a growing body of literature has revealed the biophysiological mechanisms underlying the associations between social disconnectedness and chronic inflammation, which play an important role in health and longevity^43^,^44^. A recent study has also reported that the elevation of CRP could be limited by the marital status of individuals^45^,^46^. Interestingly, these findings may explain the mechanisms underlying the relationship between QoL and relapse in schizophrenia. QoL has been reported to be an independent predictor for relapse in schizophrenia, more than the severity of symptoms^47^. It may be that relapse is triggered by mechanisms associated with inflammatory processes associated with QoL changes. Taken together, these findings raise the possibility that patients with schizophrenia destined to develop chronic inflammation might be identifiable using QoL measures and might potentially benefit from adapted therapeutic strategies to prevent it. QoL could therefore be considered a reliable predictor of chronic inflammation by healthcare professionals. From this perspective, clinicians should be encouraged to think about chronic inflammation in patients reporting poor QoL.

We also hypothesise that chronic inflammation can cause changes in QoL. In accordance with this hypothesis, a recent study suggested that the progression of chronic inflammatory disease may exert a significant negative impact on QoL in both emotional and social domains^10^. This hypothesis may be supported by the sickness behavioural model^48^,^49^. CRP is a general marker of inflammatory processes and is associated with several interleukins (e.g., IL-6) functionally involved in the development of symptoms of sickness through direct effects on the central nervous system^48^. Symptoms of sickness (e.g., fatigue, reduced appetite, sleep disorders, altered mood and cognition) are well known to negatively impact QoL amongst non-psychiatric populations^48^. Our results suggest that symptoms of sickness may also be related to poor QoL in patients with schizophrenia. Future studies should confirm this hypothesis and then explore the beneficial value of various interventions including pharmaceutical and non-pharmaceutical strategies on chronic inflammation, the symptoms of sickness and QoL in patients with schizophrenia.

The association between educational level, BMI, smoking and CRP were expected, confirming the findings of most previous studies. A low socioeconomic standing including low education level has been found to be associated with chronic inflammation^39^,^50^,^51^. Smoking has been associated with many cardiovascular risk factors, including high CRP levels, which remains in former smokers^52^,^53^. By producing oxidative stress and stimulating hematopoietic cells, smoking activates pro-inflammatory cytokines (e.g., IL-6), which in turn stimulate the production of CRP^52^,^54^. Lastly, an increase in macrophage infiltration in fat tissues has been observed in excess of weight, which promotes the substantial production of inflammatory cytokines^55^. In contrast, the lack of an association between CRP and symptoms is more surprising. However, previous studies have reported discordant results on this issue^17^,^19^. The inconsistencies may be because possible confounding factors (e.g., BMI, smoking, alcohol consumption and antipsychotic medications) were rarely considered in previous studies^56^.

### Limitations and perspectives

Several limitations should be considered in our study

First, an important methodological problem is the definition of our groups (normal CRP level and high CRP). Indeed, there are no generally accepted criteria for relapse. However, we have chosen the most consensual definition in the recent scientific literature, recognized as the cut-off point for high cardiovascular risk^21^,^35^,^36^.

Second, CRP was the sole marker of inflammation in this study. Although CRP is strongly associated with IL-6 activity, we did not directly assess any of the cytokines. Future studies with extensive assessment of inflammatory markers may be required.

Third, we have only collected CRP at one time point, and repeated testing has been recommended to confirm elevated plasma levels^35^ because concentrations can be affected by acute infection. However, patients with acute infections were removed and did not alter any of the results.

Last, although our work accounts for a large set of potentially confounding variables, additional factors might have been interesting to consider. Particularly, cognitive impairment, a core symptom of schizophrenia, is associated with metabolic syndrome^57^,^58^ and chronic inflammation^19^. However, as most studies revealed nonsignificant relationships between most neurocognitive measures and QoL^59^,^60^, our findings should not be modified.

## Conclusion

Our study suggests that chronic inflammation is moderatly associated with lower QoL levels in patients with schizophrenia. After replications with longitudinal approaches, the association between QoL and chronic inflammation may offer interesting interventional prospects to act both on inflammation and QoL in patients with schizophrenia. Moreover, it would be important to include systematic measures of inflammation markers into future clinical trials or intervention studies in schizophrenia (including cognitive or social interventions) in order to get a broader insight on the effect of those interventions which may not only concern clinical symptoms and quality of life but also inflammation markers. Such intervention studies may also be relevant to examine the dynamics of the relationship between clinical symptoms, QoL and inflammation markers.

## Additional Information

**How to cite this article**: Faugere, M. *et al*. Quality of life is associated with chronic inflammation in schizophrenia : a cross-sectional study. *Sci. Rep*. **5**, 10793; doi: 10.1038/srep10793 (2015).

## Figures and Tables

**Table 1:** Socio-demographic and clinical characteristics of the study sample (n = 256).

	Entire Group (N = 256)		Normal CRP level ≤3.0 mg (N = 156)		High CRP >3.0 mg (N = 100)		Normal CRP level vs. High CRP p-value
	Mean or %	SD	Mean or %	SD	Mean or %	SD	p
Gender (Male)	184 (71.9%)	—	116 (74.4%)	—	68 (68.0%)	—	0.270^a^
Age (years)	35.96	12.01	34.62	11.60	38.03	12.38	**0.027**
Education level (>12 years)	144 (56.3%)	—	97 (62.2%)	—	47 (47.5%)	—	**0.021**^a^
Professional activity (yes)	39 (15.2%)	—	24 (16.1%)	—	15 (15.3%)	—	0.866^a^
Duration of disorder (years)	13.04	9.72	11.86	9.31	14.82	10.11	**0.018**
Body mass index	26.63	5.35	25.33	4.93	28.68	5.35	**0.000**
Presence of atypical antipsychotic	226 (88.3%)	—	136 (87.2%)	—	90 (90.0%)	—	0.494^a^
*Presence of Clozapine*	62 (24.2%)	—	36 (23.1%)	—	26 (26.0%)	—	0.594^a^
*Presence of Olanzapine*	40 (15.6%)	—	27 (17.3%)	—	13 (13.0%)	—	0.354^a^
Chlorpromazine equivalent dose (mg/day)	856.69	791.44	844.31	899.46	875.76	591.47	0.758
Fagerström test	5.36	2.724	4.98	2.80	5.88	2.55	**0.041**
AUDIT^b^	6.33	7.76	6.60	8.09	5.99	7.34	0.627
PANSS^c^ *Total*	71.09	24.40	70.33	25.16	72.27	23.24	0.535
PANSS *Positive factor*	15.20	6.88	14.82	7.07	15.79	6.56	0.272
PANSS *Negative factor*	20.00	7.95	19.95	8.14	20.08	7.69	0.898
PANSS *General psychopathology factor*	35.89	12.09	35.56	12.46	36.40	11.54	0.588
SQoL 18^d^ index	54.87	19.14	57.22	19.35	51.27	18.32	**0.016**
Psychological Well-being	57.60	28.72	60.20	30.03	53.62	26.25	0.076
Self-Esteem	55.88	30.48	58.72	29.97	51.52	30.89	0.067
Family Relationships	62.55	27.58	64.14	26.40	60.10	29.27	0.257
Relationships with Friends	42.83	31.10	43.09	30.54	42.42	32.08	0.868
Resilience	58.93	27.09	61.40	27.99	55.13	25.33	0.073
Physical Well-being	56.82	26.37	60.77	23.20	50.76	29.71	**0.005**
Autonomy	62.05	28.20	62.66	28.26	61.11	28.23	0.671
Sentimental Life	42.33	29.74	46.79	30.40	35.48	27.47	**0.003**

^a^χ2 test for qualitative variables

^b^Alcohol Use Disorders Identification Test

^c^Positive And Negative Syndrome Scale

^d^Schizophrenia Quality of Life questionnaire 18: Scores range from 0 to 100; higher scores represent higher QoL.

**Table 2:** Factors associated with high CRP: multivariate analysis.

Factors	Adjusted odds ratio	95%CI^a^	p-value
Gender (/Male)	0.50	0.17-1.49	0.216
Age (years)	1.03	0.97-1.09	0.291
Education level (/>12 years)	4.15	1.55-11.07	0.005
Presence of atypical antipsychotic (/Absence)	0.50	0.13-1.97	0.322
Duration of disorder (years)	1.01	0.94-1.09	0.794
Body mass index	1.16	1.06-1.28	**0.002**
Fagerström Test	1.22	1.01-1.47	**0.043**
AUDIT^b^	1.01	0.95-1.07	0.721
PANSS^c^ total	0.99	0.97-1.01	0.392
SQoL 18^d^ index	0.97	0.94-0.99	**0.021**

^a^Confidence Interval

^b^Alcohol Use Disorders Identification Test

^c^Positive And Negative Syndrome Scale

^d^Schizophrenia Quality of Life questionnaire 18

**Table 3:** SQoL 18 dimensions scores associated with high CRP: multivariate analyses.

SQoL 18^a^ dimensions	Adjusted odds ratio	95%CI^b^	p-value
Psychological Well-being	0.98	0.96-0.99	**0.020**
Self-Esteem	0.99	0.97-1.00	0.082
Family Relationships	1.00	0.98-1.02	0.995
Relationships with Friends	0.99	0.98-1.01	0.329
Resilience	0.99	0.97-1.00	0.110
Physical Well-being	0.97	0.95-0.99	**0.005**
Autonomy	0.99	0.98-1.01	0.534
Sentimental Life	0.98	0.97-0.99	**0.015**

^a^Schizophrenia Quality of Life questionnaire 18: scores range from 0 to 100; higher scores represent higher QoL.

^b^Confidence Interval.
